# Fluid therapy and outcome: a prospective observational study in 65 German intensive care units between 2010 and 2011

**DOI:** 10.1186/s13613-018-0364-z

**Published:** 2018-02-17

**Authors:** Christian Ertmer, Bernhard Zwißler, Hugo Van Aken, Michael Christ, Fabian Spöhr, Axel Schneider, Robert Deisz, Matthias Jacob

**Affiliations:** 1 Department of Anaesthesiology, Intensive Care and Pain Medicine, University Hospital Münster, 48149 Münster, Germany; 2 Department of Anaesthesiology, University Hospital, LMU Munich, 80337 Munich, Germany; 3 Department of Emergency and Critical Care Medicine, Paracelsus Medical University, 90419 Nuremberg, Germany; 4 Department of Anaesthesiology and Intensive Care Medicine, Sana Kliniken Stuttgart, 70174 Stuttgart, Germany; 5 Department of Anaesthesiology and Intensive Care Medicine, University of Cologne, 50937 Cologne, Germany; 6 Department of Anaesthesiology, Krankenhaus Barmherzige Brueder, 54292 Trier, Germany; 7 Department of Intensive Care and Intermediate Care, RWTH University Hospital Aachen, 52074 Aachen, Germany; 8 Department of Anaesthesiology, Surgical Intensive Care and Pain Medicine, St. Elisabeth Hospital, St.-Elisabeth-Str. 23, 94315 Straubing, Germany

**Keywords:** Fluid therapy, Critical illness, Colloids, Crystalloids, Hydroxyethyl starch, Acute kidney injury

## Abstract

**Background:**

Outcome data on fluid therapy in critically ill patients from randomised controlled trials may be different from data obtained by observational studies under “real-life” conditions. We conducted this prospective, observational study to investigate current practice of fluid therapy (crystalloids and colloids) and associated outcomes in 65 German intensive care units (ICUs). In total, 4545 adult patients who underwent intravenous fluid therapy were included. The main outcome measures were 90-day mortality, ICU mortality and acute kidney injury (AKI). Data were analysed using logistic and Cox regression models, as appropriate.

**Results:**

In the predominantly post-operative overall cohort, unadjusted 90-day mortality was 20.1%. Patients who *also* received colloids (54.6%) had a higher median Simplified Acute Physiology Score II [25 (interquartile range 11; 41) vs. 17 (7; 31)] and incidence of severe sepsis (10.2 vs. 7.4%) on admission compared to patients who received *exclusively* crystalloids (45.4%). 6% hydroxyethyl starch (HES 130/0.4) was the most common colloid (57.0%). Crude rates of 90-day mortality were higher for patients who received colloids (OR 1.845 [1.560; 2.181]). After adjustment for baseline variables, the HR was 1.666 [1.405; 1.976] and further decreased to indicate no associated risk (HR 1.003 [0.980; 1.027]) when it was adjusted for vasopressor use, severity of disease and transfusions. Similarly, the crude risk of AKI was higher in the colloid group (crude OR 3.056 [2.528; 3.694]), after adjustment for baseline variables OR 1.941 [1.573; 2.397], and after full adjustment OR 0.696 [0.629; 0.770]), the risk of AKI turned out to be reduced. The same was true for the subgroup of patients treated with 6% HES 130/0.4 (crude OR 1.931 [1.541; 2.419], adjusted for baseline variables OR 2.260 [1.730; 2.953] and fully adjusted OR 0.800 [0.704; 0.910]) as compared to crystalloids only.

**Conclusions:**

The present analysis of mostly post-operative patients in routine clinical care did not reveal an independent negative effect of colloids (mostly 6% HES 130/0.4) on renal function or survival after multivariable adjustment. Signals towards a reduced risk in subgroup analyses deserve further study.

*Trial registration* ClinicalTrials.gov Identifier: NCT01122277, registered May 11th, 2010

**Electronic supplementary material:**

The online version of this article (10.1186/s13613-018-0364-z) contains supplementary material, which is available to authorized users.

## Background

Fluid therapy in critically ill patients is an important issue especially in initial stabilisation [1, 2]. The optimal fluid during the first “golden” hours remains controversial [3, 4]. Comparing different fluids prospectively regarding survival and organ failure of hypovolemic patients is difficult, since transfer to the participating intensive care units (ICUs), obtaining consent, randomisation and preparing study fluids take time. Recent trials relating the use of hydroxyethyl starch (HES) in critically ill patients to negative outcomes [5–8] largely suffered from this problem: identifying participants to first infusion of study fluid took up to 24 h. Therefore, due to sufficient initial (pre-study) resuscitation [9], most patients were already stabilised at inclusion [10]. Thus, these studies did not compare crystalloids versus colloids for resuscitation, but for maintenance [5, 7, 8, 11]. Moreover, results in sepsis have been extrapolated to all patients with fluid depletion [12], suspecting harm in, for example, perioperative patients, although this is not supported by current evidence. In contrast, timing and indication for fluid therapy in early resuscitation appear to be decisive for harm or benefit. This may have contributed to the decision of the European Medicines Agency (EMA) to differentiate between specific disease entities.

The present prospective, non-interventional multicentre registry aimed at gaining data on the practice of fluid therapy and associated outcomes. The goals were to assess the impact of colloids per se, but also specific colloids on 90-day survival, ICU mortality and acute kidney injury (AKI). Notably, all data were obtained prior to the EMA decision on HES solutions in 2013.

## Methods

### Aim

The aim of this study is to gather data on the practice of fluid therapy and associated outcomes in order to assess the impact of specific colloids and colloids in general on 90-day survival, ICU mortality and acute kidney injury (AKI).

### Study design

RaFTinG (Rational Fluid Therapy in Germany) is a prospective, observational, multicentre database. It assessed the characteristics of unselected ICU patients, focusing on fluid therapy and related outcomes. For recruitment, all German ICUs received an invitation letter and a notification in “Deutsches Ärzteblatt”.

### Setting

Sixty-five German ICUs participated in this registry.

### Study population

Patients with an indication for fluid therapy (judged by the attending physician) and presumed length of ICU stay > 24 h were included. Exclusion criteria were age < 18 years, psychological disorders, reasonable doubt regarding the patient’s discernment and institutionalisation upon court or other official order. Inclusion started 01.06.2010 and ended 31.05.2011. Centres were offered four inclusion schemes to avoid selective inclusion: 1. all patients, 2. all patients admitted on a specific weekday, 3. all patients admitted in one week per month and 4. first 10 consecutive patients per month.

### Study protocol and collected data

No specifications regarding diagnostics, medication or procedures were made. All relevant decisions were performed as part of usual care. Only routine records were documented for the study starting at admission to the ICU. Basic biometrical data, admission diagnoses, haemodynamic and laboratory parameters and severity scores (Acute Physiology And Chronic Health Evaluation, APACHE II; Simplified Acute Physiology Score, SAPS II; Sequential Organ Failure Assessment score, SOFA) were documented. On each ICU day, new diagnoses, haemodynamic and laboratory variables, fluid balance and therapeutic interventions were assessed. Documentation was completed by the medical condition at discharge.

ICU survivors were contacted by postal mail to retrieve survival status 90 days after ICU admission. If no reply was returned, survival status was attempted to be retraced via telephone calls and the residents’ registration offices.

Data entries were possible in electronic or paper forms (Additional file 1: Supplemental digital content 1) as preferred by the centres. Automatic enquiries for values outside of pre-specified limits ensured data validity. All data were continuously checked for formal and content-related errors. Missing and inconsistent information was reassessed.

### Outcome parameters and pre-defined subcohorts

Main outcome parameters were 90-day mortality (death within 90 days after first ICU admission), ICU mortality and AKI (RIFLE [13] “failure”). Renal replacement therapy (RRT) was analysed for completeness, despite the fact that, without protocol, it is an inaccurate parameter.

Patients were a priori stratified as having received crystalloids *and* colloids or exclusively crystalloids. Patients having received crystalloids *and* colloids were further substratified a priori as having received gelatine, HES 130/0.4, HES 130/0.42), HES 200/0.5 or human albumin. Patients who were treated with more than one type of colloid were excluded from subcohort analyses.

Data were also a priori stratified for surgical or medical patients and patients with or without severe sepsis on admission.

### Statistical analysis

Data are presented as median (25th; 75th percentiles) for numeric variables or percentages for categorical variables, if not otherwise specified. Crude results cover the whole study population. Univariate data comprise only patients eligible for multivariable analysis. The predicted individual risk of mortality on admission was calculated from SAPS II and APACHE II scores [14, 15]. We used the highest calculated risk for further analyses because a relevant subset of patients only had entries for one of these scores. In order to assess the maximum mortality risk for each patient, the score that predicted a higher risk was used for the regression analyses. Comparison of predicted vs. actual mortality shows that both are well correlated (Additional file 1: Supplemental digital content 2).

For all multivariable tests, covariables with a clear clinical relevance on the investigated outcome were chosen as cofactors and restricted to those with a *p* < 0.10 in univariate analysis in the model. Colloid dose is entered as a continuous variable in mL into the statistical models.

For AKI, RRT and ICU mortality, a multiple logistic regression model was fitted with adjustment for the predicted mortality risk in percentile steps of 10% (resulting in an ordinal covariable stratifying the predicted risk from 0–10 up to 90–100%), gender, chronic kidney disease (CKD, according to KDOQI [16]) and severe sepsis (according to ACCP/SCCM [17]) on admission as categorical cofactors (model referred to as “baseline adjustment” in the tables).

To enhance structural equality of the cohorts, the covariables obtained at ICU admission and variables derived during ICU stay (AUC of SOFA score until event or end of stay, cumulative volume of red blood cell products, cumulative volume of other blood products, cumulative fluid balance, application of vasopressor equivalent > 0.6 mg/h and daily crystalloid infusion) were included as covariables in a second analysis (referred to as “full model”).

Association of covariates was tested for significance and removed when not significant to reduce the number of model parameters to be estimated.

Norepinephrine equivalent was defined as 1 mg norepinephrine being equivalent to 1 mg epinephrine or 100 mg of dopamine [18].

90-day survival was analysed by Cox regression with the cofactors and covariables given above. To account for missing follow-up data, the following approaches were chosen: (1) include only patients with complete follow-up data; (2) assume that all patients with unknown status die 1 day after discharge (“worst case”); (3) assume that 10% of all patients die after discharge (unknown status is extrapolated based on post-ICU mortality); (4) assume that 28% of all patients die after discharge (unknown status is extrapolated based on mortality in the cohort without 90-d follow-up); (5) assume that all patients with unknown status survive until 90 days post-discharge (“best case”).

For the multivariable analyses, hazard ratios (HRs) were used to describe point estimates of the instantaneous risk ratio between cohorts in Cox regression analysis. Odds ratios (ORs) were used to quantify the cumulative risk estimate presented derived from logistic regression.

Factors with an exploratory p value of less than 0.05 in the regression equation were considered relevant for the event under investigation. The impact of specific fluids in each analysis is shown as the adjusted odds ratio or hazard ratio and the respective 95% confidence intervals [19], keeping all other covariables constant.

All statistical analyses were done with SAS version 9.3.

## Results

### Participating centres and patient recruitment

**Fig. 1:**
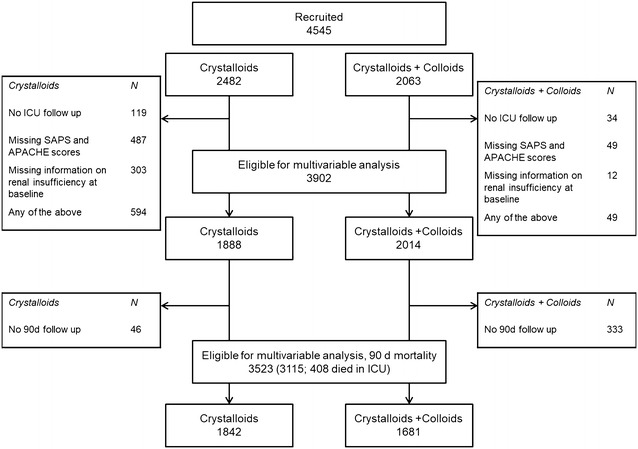
Patient flow

Sixty-five study centres documented 4545 patients. 70.9% (3223) of patients were admitted from the operating theatre. In total, 3902 (85.9%) had records for each adjustment variable and were valid for multivariable analysis (Fig. 1). Baseline characteristics are presented in Table 1. Patients with higher severity of illness (SAPS II and APACHE II scores) or with severe sepsis on admission were more likely to have received colloids later on. An overview about primary diagnoses of the patient populations is presented in Additional file 1: Supplemental digital content 3.

**Table 1:** Baseline characteristics of study patients and subcohorts

	All patients entered into database				Only patients eligible for multivariable analysis				*p* value
	All fluids	Crystalloids only	Crystalloids + colloids	Crystalloids + HES 130/0.4	All Fluids	Crystalloids only	Crystalloids + colloids	Crystalloids + HES 130/0.4	Crystalloids vs. colloids (all/eligible for multivariable analysis)
*N* (*n* [%])	4545 [100]	2482 [54.6]	2063 [45.4]	1175 [25.9]	3902 [100]	1888 [48.4]	2014 [51.6]	1128 [28.9]	
Age (years)	68 [55; 76]	68 [55; 76]	68 [56; 75]	68 [55; 76]	68 [55; 76]	68 [55; 76]	68 [56; 75]	68 [55;76]	0.784/0.641^b^
Gender, male (*n* [%])	2788 [61.3]	1491 [60.1]	1297 [62.9]	761 [64.8]	2392 [61.3]	1123 [59.5]	1269 [67.2]	730 [64.7]	0.058/0.026^c^
Admission type: surgical (*n* [%])	3223 [70.9]	1714 [69.1]	1509 [73.1]	986 [83.9]	2782 [71.3]	1316 [69.7]	1466 [72.8]	955 [84.7]	0.003/0.036^c^
Cardiac surgery (*n* [%])	686 [15.1]	310 [12.5]	376 [18.2]	351 [29.9]	659 [16.9]	290 [15.4]	369 [18.3]	348 [30.9]	<0.001/0.015^c^
Severe sepsis on admission (*n* [%])	394 [8.7]	184 [7.4]	210 [10.2]	71 [6.0]	370 [9.5]	163 [8.6]	207 [10.3]	71 [6.3]	0.001/0.090^c^
History of CKD (*n* [%])	828 [18.2]	449 [18.1]	379 [18.4]	161 [13.7]	622 [15.9]	312 [16.5]	310 [16.4]	144 [12.4]	0.837/0.356^c^
APACHE II	20 [14, 25]	18 [13, 23]	22 [17, 27]	24 [19, 30]	20 [14, 25]	18 [13, 23]	22 [17, 27]	24 [19, 30]	<0.001/< 0.001^b^
SAPS II	21 [9; 36]	17 [7, 31]	25 [11; 41]	34 [18; 52]	21 [9; 36]	17 [8, 31]	25 [11; 41]	34 [18; 52]	<0.001/< 0.001^b^
Probability of mortality [%]^a^	39.0	33.3	44.7	50.7	39.1	33.1	44.7	51.0	<0.001/< 0.001^b^
AKI (RIFLE “failure”) (*n* [%])	560 [12.3]	174 [7.0]	386 [18.7]	179 [15.2]	549 [14.1]	167 [8.8]	382 [19]	178 [15.8]	<0.001/< 0.001^c^
Renal replacement therapy in ICU (*n* [%])	361 [7.9]	78 [3.1]	283 [13.7]	133 [11.3]	358 [9.2]	76 [4]	282 [14]	133 [11.8]	<0.001/< 0.001^c^
ICU mortality (*n* [%])	408 [9.3]	129 [5.5]	279 [13.8]	119 [10.2]	367 [9.4]	92 [4.9]	275 [13.7]	118 [10.5]	<0.001/< 0.001^c^
Length of ICU stay (days)	3 [1, 9]	2 [1, 5]	6 [3, 12]	5 [2, 11]	4 [2, 8]	2 [1, 5]	6 [3, 13]	5 [3, 11]	<0.001/< 0.001^b^
90-day mortality (*n* [%])	707 [20.1]	284 [15.4]	423 [25.2]	191 [18.8]	613 [22.4]	198 [14.6]	415 [30.1]	187 [21.9]	<0.001/< 0.001^c^

Data are given as absolute number and percentage or median [25th; 75th percentiles], as appropriate

*AKI* acute kidney injury, *APACHE* acute physiology and chronic health evaluation score, *CKD* chronic kidney disease, *ICU* intensive care unit, *SAPS* simplified acute physiology score

^a^Probability of mortality was calculated from APACHE II and SAPS II scores as given in methods

^b^Wilcoxon–Mann–Whitney test

^c^Chi-squared contingency table test

### Fluid therapy

During ICU stay, 54.6% (2482) of patients received *exclusively* crystalloids, whereas 45.4% (2063) received crystalloids *and* colloids. 57.0% (1175) of the latter collective received 6% HES 130/0.4 (Table 1). Considerably less patients were treated with other colloids (6% HES 130/0.42, gelatine and human albumin) and were therefore excluded from subgroup analyses (Additional file 1: Supplemental digital content 4). Sixteen centres used exclusively crystalloids in all documented patients (207).

**Fig. 2:**
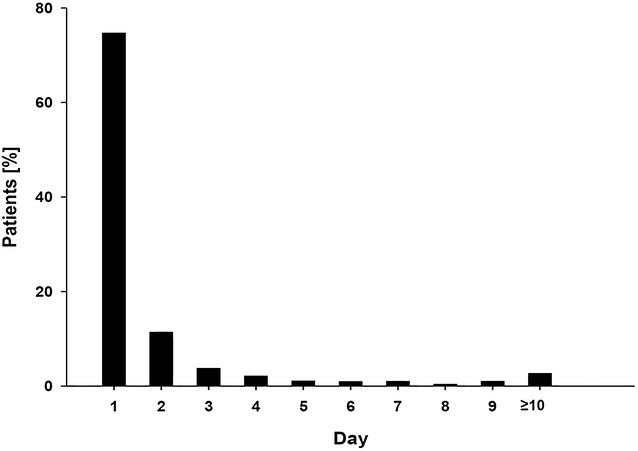
Day of first colloid infusion in study patients receiving colloids. This figure depicts the day of ICU stay on which the patients receiving colloids were infused the first dose of colloids

Fluid balances are presented in Additional file 1: Supplemental digital content 5 and 6. Colloid dose is given in Additional file 1: Supplemental digital content 7. Cumulative fluid balance and fluid balance on day 1 were more positive for patients receiving crystalloids *and* colloids as compared to sole crystalloid therapy (Additional file 1: Supplemental digital content 6). Among patients treated with colloids, 77.2% (1555 patients) received colloids on day 1 (Fig. 2), with a median amount of one unit of 500 mL [500; 1000]. Amounts of infused blood products significantly differed between cohorts, but absolute differences were very small (Additional file 1: Supplemental digital content 6).

### 90-day survival

77.5% of patients had 90-day follow-up data (3115 plus 408 who died on ICU). 55.0% (1713) of patients with follow-up data received only crystalloids and 45.0% (1402) received colloids, a ratio that is similar to the total database (54.6 vs. 45.4%).

Unknown status for 90-day follow-up was associated with lower SAPS II and APACHE II scores on admission (SAPS II median 13 [6, 26] vs. 24 [11, 38], *p* < 0.001; APACHE II median 19 [13, 24] vs. 20 [15, 26], *p* < 0.001), as well as significantly lower cumulative crystalloid infusion and red blood cell transfusions. In contrast, there was no significant association of loss to 90-day follow-up with length of ICU stay, CKD or sepsis on admission.

Overall 90-day mortality was 20.1% (707 of 3523). Crude 90-day mortality of patients who received colloids was higher than in patients treated exclusively with crystalloids (25.5% (423 of 1681) vs. 15.4% (284 of 1842) (crude OR in the overall population 1.845 [1.560; 2.181]). After adjustment for baseline covariables only and adjustment for baseline and progress variables, the adjusted risk associated with colloids decreased stepwise by multivariable Cox regression and was no longer statistically significant (Table 2). Independent risk factors were predicted mortality, female gender, severe sepsis and CKD on admission, vasopressor use, SOFA score (AUC) and cumulative fluid balance. These findings were similar in patients who received HES 130/0.4 (Additional file 1: Supplemental digital content 8). In addition, results were homogenous among the subcohorts of septic and non-septic (colloid use 0.923 [0.874; 0.974]; 1.002 [0.908; 1.106]) as well as surgical and medical patients (colloid use 0.980 [0.912; 1.053], 0.945 [0.891; 1.001]). Sensitivity analysis did not suggest that data were influenced by missing follow-up data (Additional file 1: Supplemental digital content 9).

**Table 2:** Effects of colloid infusion on 90-day mortality in Cox regression analysis

Odds/hazard ratio estimates	Unadjusted/crude mortality (population eligible for multivariable analysis)^a^			Adjustment for baseline variables			Full model, adjusted for evolution variables		
Effect	OR	95% CI		HR	95% CI		HR	95% CI	
Colloid infusion	2.027	1.697	2.422	1.666	1.405	1.976	1.003	0.980	1.027
Gender, male				1.141	0.974	1.337	0.798	0.679	0.936
Predicted risk of death 90–99%				6.003	3.773	9.551	4.505	2.781	7.298
Predicted risk of death 80–89%				3.266	2.046	5.212	2.630	1.637	4.226
Predicted risk of death 70–79%				3.018	1.905	4.780	2.250	1.412	3.586
Predicted risk of death 60–69%				2.893	1.870	4.474	2.184	1.403	3.401
Predicted risk of death 50–59%				2.171	1.365	3.453	1.718	1.070	2.757
Predicted risk of death 40–49%				2.073	1.353	3.176	1.732	1.128	2.660
Predicted risk of death 30–39%				1.648	1.064	2.552	1.484	0.957	2.303
Predicted risk of death 20–29%				1.777	1.154	2.736	1.611	1.045	2.482
Predicted risk of death 10–19%				1.833	1.189	2.826	1.759	1.140	2.712
Chronic kidney disease				2.149	1.812	2.549	1.960	1.645	2.336
Severe sepsis on admission				2.017	1.648	2.469	1.284	1.025	1.608
Vasopressor equivalent > 0.6 mg/h							1.019	1.002	1.035
SOFA score AUC							1.030	1.024	1.036
Cumulative red blood cell products (per litre)							0.977	0.958	0.997
Cumulative other blood products (per litre)							1.002	0.968	1.037
Cumulative fluid balance (per litre)							1.014	1.008	1.020
Daily crystalloid infusion (per litre)							1.121	1.067	1.178

Risk of mortality was estimated from severity scores as detailed in the methods section. Gender, risk of mortality categories, vasopressor requirement, chronic kidney disease and severe sepsis on admission were used as binary variables (yes/no), whereas the hazard ratios (HRs) for fluid-based variables and Sequential Organ Failure Assessment (SOFA) score describe the excess risk per litre of fluid and per SOFA area under the curve (AUC), respectively

*CI* confidence interval

^a^To show the effect of the modelling, we showed the unadjusted odds ratio for the population that was eligible for the multivariable analysis. Crude OR for all patients in the registry is shown in the text

### ICU mortality

Overall ICU mortality was 9.3% (408 of 4392, 153 missing data). Treatment with colloids was associated with higher crude ICU mortality compared to crystalloids only (13.8% (279 of 2029) versus 5.5% (129 of 2363), crude OR in the overall population 2.761 [2.221; 3.433]). The risk of ICU mortality associated with colloids or 6% HES 130/0.4 decreased progressively after multiple logistic regression analysis using baseline and progress covariables (Additional file 1: Supplemental digital content 10 and 11). Independent risk factors were predicted mortality, female gender, vasopressor use, severe sepsis and CKD on admission, as well as cumulative fluid balance. Notably, for patients in the subcohort without severe sepsis on admission, the adjusted risk of ICU mortality was lower for patients treated with colloids in general (crude OR 2.367 [1.539; 3.643]; multivariable adjusted OR 0.923 [0.874; 0.974]) or 6% HES 130/0.4 (crude OR 2.179 [1.223; 3.882]; multivariable adjusted OR 0.905 [0.833; 0.983]) compared to crystalloids only.

### Acute kidney injury

The overall incidence of AKI was 12.3% (560 of 4545). The crude incidence of AKI was higher in patients treated with colloids compared to crystalloids only (18.7% (386 of 2063) vs. 7.0% (174 of 2484), crude OR 3.056 [2.528; 3.694]). With multivariable logistic regression analysis, the risk associated with colloids or 6% HES 130/0.4 decreased after adjustment for baseline covariables. In the full model adjusted for baseline and progress covariables, colloids and 6% HES 130/0.4 were associated with a reduced risk of AKI (Table 3 and Additional file 1: Supplemental digital content 12). Independent risk factors were predicted mortality, severe sepsis and CKD on admission as well as SOFA score (AUC).

**Table 3:** Effects of colloid infusion in logistic regression analysis of RIFLE “failure”

Odds ratio estimates	Unadjusted/crude mortality (population eligible for multivariable analysis)^a^			Adjustment for baseline variables			Full model, adjusted for evolution variables		
Effect	OR	95% CI		OR	95% CI		OR	95% CI	
Colloid infusion	2.412	1.987	2.929	1.941	1.573	2.397	0.696	0.629	0.770
Gender, male				1.008	0.824	1.233	0.943	0.763	1.167
Predicted risk of death 90–99%				20.787	11.773	36.700	12.490	6.724	23.201
Predicted risk of death 80–89%				5.754	3.349	9.885	4.823	2.731	8.516
Predicted risk of death 70–99%				3.934	2.326	6.653	3.061	1.769	5.297
Predicted risk of death 60–69%				2.756	1.677	4.532	1.940	1.154	3.262
Predicted risk of death 50–59%				2.461	1.470	4.121	1.921	1.129	3.268
Predicted risk of death 40–49%				1.705	1.061	2.738	1.336	0.822	2.173
Predicted risk of death 30–39%				1.331	0.819	2.163	1.100	0.672	1.800
Predicted risk of death 20–29%				1.043	0.642	1.694	0.773	0.471	1.271
Predicted risk of death 10–19%				1.237	0.763	2.005	1.041	0.639	1.696
Chronic kidney disease				11.089	9.008	13.652	10.445	8.409	12.975
Severe sepsis on admission				8.415	6.841	10.353	11.618	9.062	14.894
Vasopressor equivalent > 0.6 mg/h							0.957	0.891	1.029
SOFA score AUC							1.046	1.039	1.053
Cumulative red blood cell products (per litre)							0.976	0.923	1.032
Cumulative other blood products (per litre)							1.208	0.988	1.476
Cumulative fluid balance (per litre)							1.011	0.994	1.029
Daily crystalloid infusion (per litre)							0.843	0.792	0.896

Risk of mortality was estimated from severity scores as detailed in the methods section. Gender, risk of mortality categories, vasopressor requirement, chronic kidney disease and severe sepsis on admission were used as binary variables (yes/no), whereas the odds ratios (ORs) for fluid-based variables and Sequential Organ Failure Assessment (SOFA) score describe the excess risk per litre of fluid and per SOFA area under the curve (AUC), respectively

*CI* confidence interval

^a^To show the effect of the modelling, we showed the unadjusted OR for the population that was eligible for the multivariable analysis. Crude OR for all patients in the registry are shown in the text

Further information about renal function and failure (incidences of AKI and RRT in patients with and without underlying CKD on admission; creatinine and diuresis) is given in Additional file 1: Supplemental digital content 13 and 14.

### Renal replacement therapy

During ICU stay, 7.9% (361) of the patients received RRT (see also Table 1). The crude incidence of RRT was considerably higher in patients treated with colloids compared to those without (13.7% (283 of 2063) vs. 3.1% (78 of 2484), crude OR for the overall population 4.904 [3.789; 6.348]). With multivariable logistic regression analysis, the risk associated with colloids or 6% HES 130/0.4 decreased after adjustment for baseline covariables. In the full model adjusted for baseline and progress covariables, colloids and 6% HES 130/0.4 were associated with a reduced risk of RRT (Additional file 1: Supplemental digital content 15). Independent risk factors for RRT included predicted risk of death, severe sepsis and CKD, AUC of the SOFA score and cumulative fluid balance. In contrast, vasopressor use was negatively associated with RRT.

## Discussion

In the present analysis of fluid therapy in roughly 4500 German ICU patients with predominantly post-operative admission, about half the patients were *exclusively* treated with crystalloids, whereas the remaining patients *also* received colloids (mainly 6% HES 130/0.4).

Whereas crude and unadjusted analyses suggested an association of colloids with adverse outcome, a stepwise adjustment for baseline and progress covariables indicates that the use of colloids per se did not affect the risk of mortality. Moreover, the association of colloids with AKI disappeared after multivariable adjustment. In subgroup analyses, the adjusted risk of AKI was lower in patients treated with colloids per se or HES 130/0.4. In the subcohort of patients admitted without severe sepsis, there was also a trend towards reduced ICU mortality with colloids per se or HES 130/0.4. The most important finding of the present study is that the effects of colloids turned from seemingly adverse in the raw data to neutral or potentially beneficial after multivariable analyses. This contradicts findings from randomised controlled trials (RCTs). One main reason for this may be the timing and dose of colloid use being different from the RCTs as detailed below.

RaFTinG is the largest database comparing crystalloids versus colloids in clinical routine for renal and overall outcome. Demographic characteristics indicate its population to be representative for German and international ICUs [20, 21]. Incidence and mortality of severe sepsis were comparable to that observed in a German epidemiological trial [20] but lower than in the Sepsis Occurrence in Acutely Ill Patients (SOAP) study [22].

The present data demonstrated colloids to be reserved for more severely ill patients with risk factors for mortality or AKI beyond the type of i.v. fluids. It is not surprising that the crude incidences of AKI and mortality were higher in “colloid” patients, since the present study was no RCT. Therefore, the observational design of RaFTinG required adjustment for confounders with impact on patient outcome.

Our approach for adjustment included the baseline variables gender, severity of disease (based on risk of mortality derived from SAPS II and APACHE II scores), chronic kidney disease and severe sepsis on admission, which affect outcome in the ICU. However, these variables do not reflect the disease progression on the ICU. Patients with the same baseline risk may develop in opposed directions, with some patients recovering without fluid resuscitation and others deteriorating and require crystalloids and/or colloids for haemodynamic support. Thus, we also included the following variables for adjustment in our final model: SOFA score to reflect the overall severity of organ failure over the course of the ICU stay, indicating a general deterioration of patient status, high-dose vasopressor infusion to reflect haemodynamic instability related to the vasculature as well as transfusions [23], which carry an independent risk of negative outcome. We consider the addition of these factors to the multivariate analysis as mandatory to approach the net effects of colloids on outcome. Nevertheless, it needs to be acknowledged, that the current approach also bears the risk of overadjustment, since some of the adjustment variables may also be affected by colloid infusion itself (e.g. transfusions). To make the data more transparent, we present crude data, baseline adjustment and full adjustment.

After multivariable adjustment for baseline covariables and progress variables, treatment with colloids in general practice did not appear to negatively affect survival. Colloids being still associated with adverse outcomes after adjustment for baseline variables may be explained by evolution of patients’ disease state after ICU admission with some patients improving and others deteriorating further. Since the latter patients are more likely to receive colloids, we also adjusted for variables of disease progress after ICU admission. Notably, the SOAP study used a very similar approach to adjustment in a very similar setting [24]. Surprisingly, our fully adjusted analysis suggests that, in low doses as used in the present cohort, colloids and specifically HES 130/0.4 are neutral in terms of 90-day mortality and might even be associated with reduced risks of AKI and ICU mortality in critically ill patients without severe sepsis. These adjusted results are in strong contrast to the unadjusted results and should therefore be judged with appropriate caution. Nevertheless, they are in agreement with the CRISTAL trial [25], which showed that in untreated shock from any reason initial treatment with crystalloids alone may limit survival. Furthermore, the present results indicate that trials conducted in septic patients may not be extrapolated to non-septic patients [12, 26].

The advantage of the RaFTinG registry compared to previous RCTs [5, 7, 8, 25] is that colloids or crystalloids infusion was based exclusively on the clinical scenario, without being influenced by study protocols. The latter do often not reflect “real-life” fluid therapy, as has been demonstrated previously [5, 7, 8]. However, this also represents a major weakness, since the study cohorts are markedly different and the statistical analysis is sophisticated. Nevertheless, the present data give an estimate of the current use and dosing of fluid therapy and its changes throughout the ICU stay. This is important, as most positive and negative effects seen with colloids and fluids per se depend on timing and dosage [1, 9]. Therefore, RaFTinG not only adds relevant data but also helps to separate clinically relevant from artificial effects and allows the design of appropriate control groups for future RCTs. In this study, colloid use was completely different from that in VISEP [5], 6S [8] or CHEST [7]. Colloids were mainly given during the first day of ICU stay, with a consistent decline thereafter. When the interventional period of recent trials began, less than 60% of the RaFTinG population still received colloids. Furthermore, patients received median volumes of only 500 mL daily, which is considerably less than the amount that patients received on average per day in previous trials, e.g. 6S (1000–1500 mL) [8]. Neither physiology nor clinical practice randomises patients for several days into “colloid” and “crystalloid receivers”. Rather, the decision to infuse the drug “fluid” should be the result of a careful and permanently re-evaluated individual assessment of the expected benefit versus the potential risk.

The current discussion about colloids, especially on the safety of HES, might have caused significant indication bias by some investigators. For example, severely ill patients may be prone to receive more colloids as their cardiac preload is thought to be more compromised. Additionally, in patients with renal impairment, some physicians might prefer gelatine, according to the results of mainly one clinical trial [27], or sole crystalloids instead of HES. Indeed, CKD at baseline was highest in patients who received gelatine in RaFTinG.

Most patients in our study were post-operative without severe sepsis on admission (Table 1). Many patients in the RCTs suggesting negative effects of HES solutions, by contrast, were admitted to the ICU due to severe sepsis [5, 7, 8]. For patients in the perioperative setting, there is no evidence for harm with the use of 6% HES 130/0.4 [28, 29] or HES solutions in general [30] from the recent literature.

The results of the present multivariable analysis are in accordance with the previous literature on 6% HES 130/0.4 in non-septic and perioperative patients, which does not suggest an adverse effect on kidney function or survival.

### Limitations of the present study

Our study has several limitations. Since it is observational, unblinded outcome assessment is unavoidable. Patients were not randomised and the cohorts are heterogeneous, with significant imbalances at baseline (e.g. severity scores, prevalence of sepsis) and many potential confounders on outcome. Even though we are confident that we were able to identify most of them and perform an appropriate adjustment, several approaches to adjusting the data are possible. It appears virtually impossible to account for the different severity of illness in the colloid cohort versus the crystalloid cohort without using adjustment parameters that are also influenced by disease progress. Thus, both risks of residual confounding and overadjustment exist. As a consequence, the adjusted results should be interpreted with caution. However, the small adjusted confidence intervals around or below 1 strongly suggest neutrality of the investigated colloids in terms of mortality and AKI. Unfortunately, although the analysis plan for the major endpoints (AKI, 90-day and ICU mortality) was designed a priori, no statistical analysis plan has been pre-published, which would have further strengthened the present results.

The present study did not investigate pre-admission fluid therapy or haemodynamics and can therefore not provide an estimate of the indication and effectiveness, which would have required a different study design [31]. Nevertheless, the timing of colloid infusion suggests colloids were used predominantly for initial or post-operative resuscitation (with pre-ICU fluid therapy, e.g. in the OR, being a blind spot).

Subcohorts having received other colloids than HES 130/0.4 were small compared to HES 130/0.4 or crystalloids. Any comparison between these subgroups must be done with caution, if at all.

Furthermore, the incidence of RRT may be a unreliable outcome measure, since most centres had no clear protocol for it. Any conclusion based on RRT should therefore be drawn with great caution.

It also needs to be acknowledged that an estimated mortality risk calculation by combining APACHE II and SAPS II (as available) may be less accurate than having a full set of both scores.

Finally, the follow-up might be considered incomplete. Besides that, missing information was not evenly distributed between both analysed groups with more incomplete baseline data with crystalloids only and more incomplete 90-day follow-up with colloids. The reason for this imbalance in missing data remains unknown, although we found that patients lost to follow-up were less severely ill on ICU admission. It is unclear how this finding may have led to a lower follow-up rate in the colloid cohort. Therefore, it is possible that unknown confounders influenced follow-up rates in the two cohorts. Nevertheless, a 90-day follow-up rate of 77.5% appears to be reasonably high when compared to 50–80% in other epidemiological cohort studies [32].

We also checked the effect of missing data on our full multivariable analysis with a best-/worst-case scenario. As expected, all risks were “diluted” by the assumption that all patients without follow-up had died. However, the risk associated with colloid use did not increase by this approach although colloid use was associated with greater loss to 90-day follow-up. Thus, the best-case/worst-case analysis suggests that missing follow-up data did not substantially affect the overall result.

## Conclusions

The present analysis of mostly post-operative patients in routine clinical care did not reveal an independent negative effect of colloids (mostly HES 130/0.4) on AKI or survival after multivariable adjustment. Contrasting results compared to published RCTs may be explained by differences in dose and duration of colloid infusion. Signals towards a reduced risk in non-septic, perioperative patients deserve further study.

## Additional file

**Additional file 1.** Case report form and additional analyses.

## Abbreviations

AKI: acute kidney injury
AUC: area under the curve
HES: hydroxyethyl starch
ICU: intensive care unit
OR: odds ratio
RCT: randomised controlled trial
CKD: chronic kidney disease
