# Supplementary material for: Fluid therapy and outcome: a prospective observational study in 65 German intensive care units between 2010 and 2011
Source: Ann Intensive Care. 2018 Feb 17;8:27. doi: 10.1186/s13613-018-0364-z (PMC5816732; doi:10.1186/s13613-018-0364-z)
Supplement: Supplementary file 1 — Additional file 1. Case report form and additional analyses. [file 13613_2018_364_MOESM1_ESM.pdf]

Supplemental digital content

Supplemental digital content 1: Case report form.

Supplemental digital content 2: Predicted vs. actual mortality, valid entries for SAPS II and APACHE II scores.

Supplemental digital content 3: Primary diagnosis of study patients, OPS-codes.

Supplemental digital content 4: Baseline characteristics and fluid data for low sample size subcohorts

Supplemental digital content 5: Daily fluid balance in study patients.

Supplemental digital content 6: Fluid balances and infusion amounts of study patients eligible for multivariable analysis and subcohorts.

Supplemental digital content 7: Daily colloid infusion in study patients

Supplemental digital content 8: Effects of colloid infusion (HES 130/0.4) in Cox regression analysis of 90-day mortality.

Supplemental digital content 9: Missing data analysis in Cox regression analysis of 90-day mortality for crystalloids vs. colloids

Supplemental digital content 10: Effects of colloid infusion in logistic regression analysis of intensive care unit mortality.

Supplemental digital content 11: Effects of colloid infusion (HES 130/0.4) in logistic regression analysis of intensive care unit mortality.

Supplemental digital content 12: Effects of colloid infusion (HES 130/0.4) in logistic regression analysis of RIFLE “failure”.

Supplemental digital content 13: Urine output and creatinine concentrations

Supplemental digital content 14: Acute kidney injury in study patients with or without pre-existing chronic kidney disease

Supplemental digital content 15: Effects of colloid infusion in logistic regression analysis of renal replacement therapy.

Aufnahmebogen - Register RaFTinG

RaFTinG

Stammdaten:

Patienten-ID: ______ Klinik-Nr.: ______

Aufnahmedatum: __ . __ . ____ Geburtsdatum: __ . __ . ____

Geschlecht: M W Größe: _ , __ m Anamnest. Gewicht: ___ kg

Übernahme von: ☐ Notarzt ☐ OP ☐ Normal ☐ Intensivstation (Verlegung von: ______)

ICD-10-Leitdiagnose bei Aufnahme auf ITS: ______

J N Teilnehmer an Volumentherapie-Studie

Relevante Nebendiagnosen

**ZNS:**	N	J N Akuter Schlaganfall: ☐ Hämorrhagischer ☐ Ischämischer	☐ TIA o. PRIND<br>J N Akutes SHT<br>J N Akute Rückenmarksläsionen	Sonstiges:
**Lunge:**	N	J N COPD, vorbestehend<br>J N Asthma, vorbestehend	J N ALI/ARDS<br>J N Ateminsuffizienz	J N Beatmung (seit:)<br>Sonstiges:
**Kardiovask. System:**	N	J N Chron. Herzinsuffizienz: NYHA ☐ I ☐ II ☐ III ☐ IV<br>J N Akut dekomp. Herzinsuffizienz	J N Arterielle Hypertonie, vorbest.<br>J N KHK, vorbestehend<br>J N ACS	Sonstiges:
**Leber:**	N	J N Akute Leberfunktionsstörungen<br>J N Ikterus, vorbestehend	J N Leberfunktionsstörungen, vorbest.<br>J N Zirrhose, vorbestehend	Sonstiges:
**Blutgerinnung:**	N	J N Antikoagulation<br>J N Thrombotisches Ereignis<br>J N Bekannte Gerinnungsstörung	J N DIC, vorbestehend<br>J N Akute Blutung<br>J N Lysetherapie	Sonstiges:
**GI-Trakt:**	N	J N Aszites<br>J N Bauchoperation	J N Ileus	Sonstiges:
**Niere:**	N	J N Chronische Niereninsuffizienz<br>J N Nierenersatzverfahren	J N Nierentransplantation<br>J N Akutes Nierenversagen	Sonstiges:
**Stoffwechsel:**	N	J N Diabetes mellitus	J N Diabetisches Koma	Sonstiges:
**Immunsystem:**	N	J N Allergien	J N Autoimmunerkrankungen	Sonstiges:
**Syst. Inflammation:**	N	☐ SIRS<br>☐ Sepsis	☐ Schwere Sepsis<br>☐ Septischer Schock	Sonstiges:
**Maligne Erkrankungen:**	N	Therapie innerhalb der letzten 3 Monate:<br>J N Chemotherapie	J N Radiotherapie	Sonstiges:
**Trauma:**	N	☐ Weichteil- oder Knochentrauma<br>☐ Polytrauma	J N Verbrennungen<br>Betroffene Körperoberfl. (in %):	Sonstiges:

Weitere wesentl. Nebendiagnosen (ICD-10): ______

**Operativer Status:**<br>N Nicht operiert	☐ Notfall-OP<br>☐ Wahleingriff	OPS-Code:<br>Datum der OP:

J N Vorgeschichte mit schwerer Organinsuffizienz und Immunschwäche

**Aufnahme-Labor:**	Na^+ (in mmol/l):<br>K^+ (in mmol/l):	Kreatinin (in mg/dl):<br>Thrombozyten (mm^{-3}):	Serum-pH:<br>Hämatokrit (in %):
**Weitere numerische Befunde:**	Temperatur (rekt. in °C):<br>MAP (in mmHg):<br>Herzfrequenz (in min^{-1}):	Atemfrequenz (in min^{-1}):<br>PaO_2 (in mmHg)*:<br>FiO_2 (in %)*:	$A\text{-}aDO_2$ (in mmHg)*:<br>*zeitgleich mit FiO_2 bzw. PaO_2 bestimmt
**Scores:**	Glasgow Coma Scale:	SAPS II:	Apache II:

Antwort senden an: Organisatorische Leitung RaFTinG
Hohlstraße 18, 69242 Mühlhausen,
Fax: 0 62 22 / 93 94 44

Datum Unterschrift

Verlaufsbogen - Register RaFTinG

RaFTinG

Stammdaten:

Patienten-ID: Klinik-Nr.:

Datum: . . Geburtsdatum: . .

Geschlecht: M W ITS-Aufnahmedatum: . .

Änderung der Leitdiagnose (ICD-10): ☐ Keine Änderung der Leitdiagnose

Weitere wesentl. Nebendiagnosen (ICD-10):

**Laborwerte:** (bei morgendlicher Visite)	Na^+ (in mmol/l):	Kreatinin (in mg/dl):
	K^+ (in mmol/l):	Kreatinin-Clear. (in ml/min):
	Ca^{2+} (in mmol/l):	Bilirubin (in mg/dl):
	Cl^- (in mmol/l):	Thrombozyten (in mm^{-3}):
	BE (in mmol/l):	Serum-pH:
	Osmolarität (in mosmol/l):	pCO_2 (in mmHg):
	Osmolalität (in mosmol/kg):	Hämoglobin (in mmol/l):
	Bicarbonat (in mmol/l):	Hämatokrit (in %):
	Laktat (in mmol/l):	
**Flüssigkeits-verluste:** (Tagesvolumen)	Urinausscheidung (in ml):	Sichtbare und messbare Blutverluste (in ml):
	Gastroösophagealer Reflux (in ml):	Tageshöchsttemperatur (in °C):
	Diarrhoe (Stuhl, in ml):	J N Schweißproduktion (>1 h)
**Flüssigkeits-zufuhr:** (Tagesvolumen)	Enterale und orale Ernährung (in l):	Parenterale Ernährung (in l):
	Produkt	Menge (in ml)
	Kristalloide:	
	Kolloide:	
	Blutprodukte:	
**Vasoaktive Substanzen:** (Maximale Zufuhr)	Dopamin (in mg/h):	Dobutamin (in mg/h):
	Adrenalin (in mg/h):	Vasopressin (in mg/h):
	Noradrenalin (in mg/h):	Terlipressin (in mg/h):
	Milrinon (in mg/h):	Enoximon (in mg/h):
**Weitere Parameter:**	min. MAP vom Vortag (in mmHg):	Temperatur (in °C):
	PaO_2 (in mmHg)*:	Gewicht (in kg):
	FiO_2 (in %)*:	J N Beatmung
	$ScvO_2$ (in %):	J N Nierenersatzverfahren
	*zeitgleich mit PaO_2 bzw. FiO_2 bestimmt	
**Scores:**	Glasgow Coma Scale: SOFA:	Core-10-TISS:
**Entlassen aus ITS:** N	Verbleib: ☐ Tod ☐ Intermediate ☐ Normalstation ☐ Verbleib unbekannt ☐ Andere ITS ☐ Reha ☐ Entlassung nach Hause	Entlassungsdatum: . . Übergabe an andere ITS: . . Sterbedatum: . . Todesursache:

Datum Unterschrift

Antwort senden an: Organisatorische Leitung RaFTinG
Hohlstraße 18, 69242 Mühlhausen,
Fax: 0 62 22 / 93 94 44

Follow-Up-Bogen – Register RaFTinG

RaFTinG

Patienten-Nr.: Klinik-Nr.:

Datum: . . Geburtsdatum: . .

Geschlecht: M W Aufnahme auf Intensivstation: . .

Adresse

Bitte füllen Sie die unten stehenden Felder für die ersten drei Monate nach Ihrem Aufenthalt auf der Intensivstation aus

Ja Nein Aus Krankenhaus entlassen

Entlassungsdatum: . .

Ja Nein Patient ist verstorben Sterbedatum: . . Todesursache:

Anschluss-behandlung:

Reha-Behandlung: ☐ ambulante Reha ☐ stationär in Reha-Klinik ☐ durch Hausarzt

Ort der Reha-Behandlung:
z.B. Praxis Dr. Mustermann, Klinik XY

Ja Nein Erneuter Klinikaufenthalt

Grund für Klinikaufenthalt:

Klinik: Abteilung:

Neue Erkrankung **nach** Klinikaufenthalt:

Ja Nein Nierenersatzverfahren (Dialyse) Dauer (Tage):

Ja Nein Besuch eines Dermatologen (Hautarzt) Grund:

Befindlichkeit:

Pflegestufe **vor** Aufenthalt auf Intensivstation:

☐ Pflegestufe 1 ☐ Pflegestufe 2 ☐ Pflegestufe 3
☐ Nicht pflegebedürftig

Pflegestufe **nach** Aufenthalt auf Intensivstation:

☐ Pflegestufe 1 ☐ Pflegestufe 2 ☐ Pflegestufe 3
☐ Nicht pflegebedürftig

Fahren Sie selbst Auto?

☐ Ja ☐ Nein ☐ Kein Führerschein

Berufstätigkeit:

☐ Berufstätig ☐ Nicht berufstätig ☐ Eingeschränkt berufstätig
☐ Vor Erkrankung bereits nicht berufstätig

Datum Unterschrift

Bitte senden Sie den ausgefüllten Bogen mit beiliegendem Freiumschlag an die Klinik zurück. Herzlichen Dank!

Supplemental digital content 2: Predicted vs. actual mortality, valid entries for SAPS II and APACHE II scores.

Supplemental digital content 2A: Predicted vs. actual ICU mortality

	Mortality in ICU					
	alive		dead		all	
	N	%	N	%	N	%
**Risk for mortality at admission**						
**missing**	508	94.8	28	5.2	536	100.0
**0 - 9%**	433	97.5	11	2.5	444	100.0
**10-19%**	598	94.6	34	5.4	632	100.0
**20-29%**	585	93.5	41	6.5	626	100.0
**30-39%**	545	93.2	40	6.8	585	100.0
**40-49%**	513	91.3	49	8.7	562	100.0
**50-59%**	257	87.7	36	12.3	293	100.0
**60-69%**	276	85.4	47	14.6	323	100.0
**70-99%**	195	84.8	35	15.2	230	100.0
**80-89%**	150	81.5	34	18.5	184	100.0
**90-99%**	80	61.5	50	38.5	130	100.0
**All**	4140	91.1	405	8.9	4545	100.0

Supplemental digital content 2B: Predicted vs. actual 90 day mortality

	90 day mortality					
	alive		dead		all	
	N	%	N	%	N	%
**Risk for mortality at admission**						
**missing**	474	88.4	62	11.6	536	100.0
**0 - 9%**	416	93.7	28	6.3	444	100.0
**10-19%**	553	87.5	79	12.5	632	100.0
**20-29%**	545	87.1	81	12.9	626	100.0
**30-39%**	511	87.4	74	12.6	585	100.0
**40-49%**	474	84.3	88	15.7	562	100.0
**50-59%**	242	82.6	51	17.4	293	100.0
**60-69%**	244	75.5	79	24.5	323	100.0
**70-99%**	173	75.2	57	24.8	230	100.0
**80-89%**	133	72.3	51	27.7	184	100.0
**90-99%**	73	56.2	57	43.8	130	100.0
**All**	3838	84.4	707	15.6	4545	100.0

Supplemental digital content 2C: Number of valid entries for SAPS II and APACHE II

		90 day mortality					
		alive		dead		all	
		N	%	N	%	N	%
SAPS II	APACHE II						
missing	missing	474	88.4	62	11.6	536	100.0
	valid	476	89.8	54	10.2	530	100.0
valid	missing	110	80.9	26	19.1	136	100.0
	valid	2778	83.1	565	16.9	3343	100.0
All		3838	84.4	707	15.6	4545	100.0

Supplemental digital content 3: Primary diagnosis of study patients.

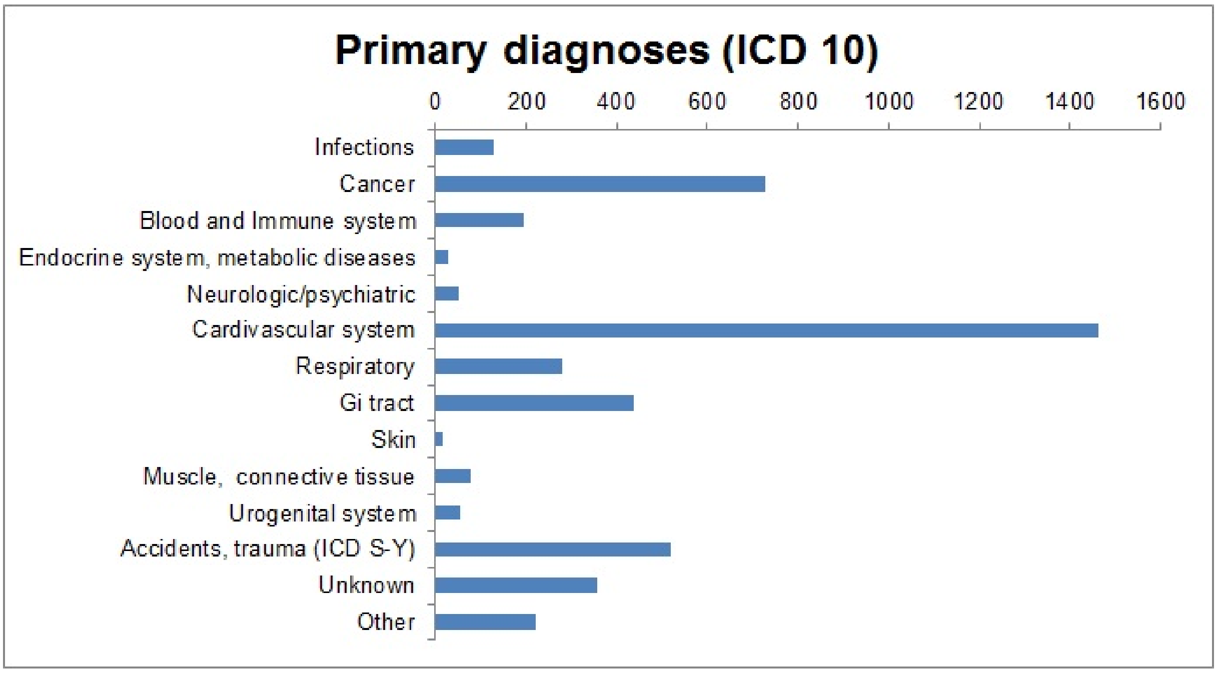

OPS-Codes for surgical patients.
405 emergency surgeries

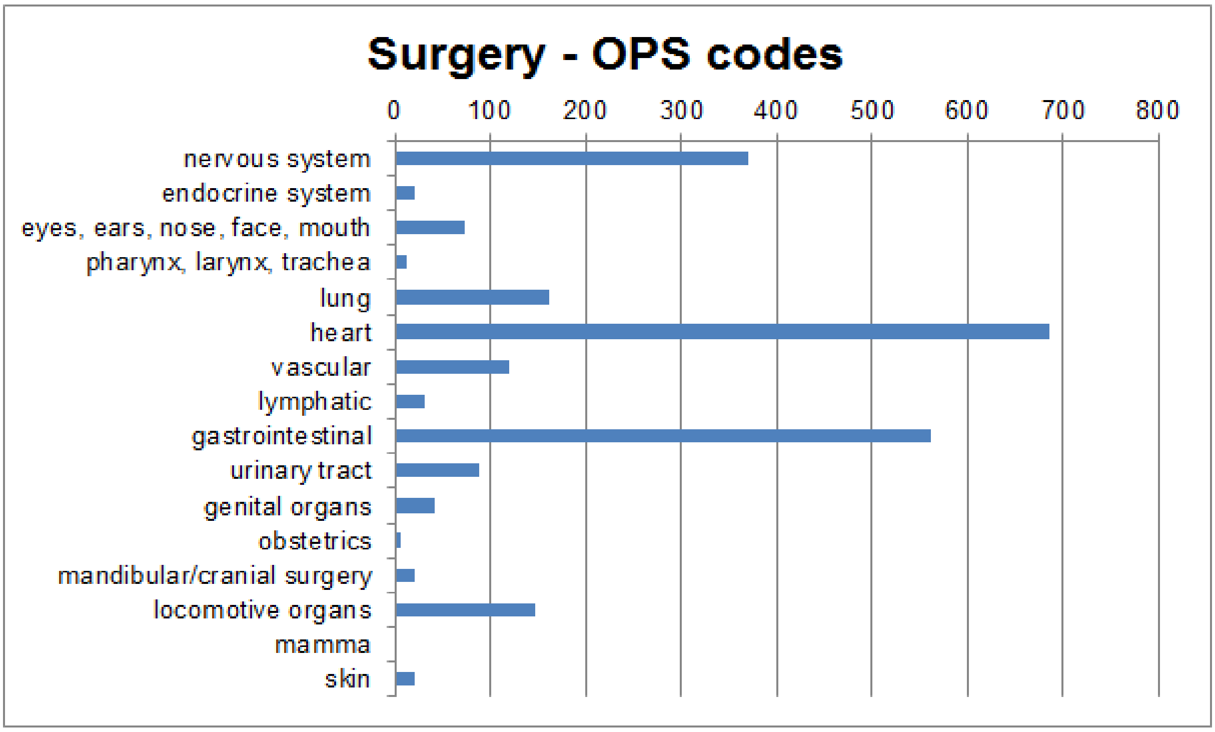

Supplemental digital content 4: Baseline characteristics and fluid data for low sample size subcohorts

Supplemental digital content 4-A: Baseline characteristics for low sample size subcohorts

	**HES 200/0.5**	**HES 130/0.42**	**Gelatin**
N (n[%])	167 [3.7]	207 [4.6]	218 [4.8]
Age (y)	68 [56; 75]	69 [56; 77]	70 [58; 75]
Gender, male (n[%])	105 [62.9]	109 [52.7]	138 [63.3]
Admission type: surgical [%]	101 [60.5]	141 [68.1]	91 [41.7]
Severe sepsis on admission (n[%])	34 [20.4]	19 [9.2]	27 [12.4]
History of CKD (n[%])	43 [25.7]	42 [20.3]	62 [28.4]
APACHE II	21 [15; 25]	19 [12; 24]	19 [13; 24]
SAPS II	12 [6; 20]	26 [9; 38]	13 [6; 25]
Mean probability of mortality [%][a]	37.8	35.7	35.3
AKI (RIFLE "failure") (n[%])	36 [21.6]	24 [11.6]	50 [22.9]
Renal replacement therapy in ICU (n[%])	21 [12.6]	12 [5.8]	37 [17.0]
ICU mortality (n[%])	33 [20.0]	32 [15.8]	24 [11.2]
Length of ICU stay (d)	5 [3; 11]	9 [3; 14]	3 [1; 9]
90-day mortality (n[%])	44 [31.9]	45 [29.0]	47 [33.6]

Supplemental digital content 4-B: Fluid data for low sample size subcohorts

	**HES 200/0.5**	**HES 130/0.42**	**Gelatin**
Cumulative fluid balance [mL per study day]	630 [237; 1212] N=167	503 [-60; 1236] N=207	990 [360; 1726] N=218
Fluid balance on day 1 [mL]	1580 [553; 2975] N=167	1690 [755; 2772] N=200	1691 [1080; 2830] N=218
Cumulative crystalloid amount [mL per study day]	2175 [1504; 2942] N=167	1735 [1275; 2649] N=207	1827 [1312; 2630] N=217
Crystalloid amount on day 1 [mL]	3100 [2175; 4163] N=165	3000 [2176; 4067] N=200	2560 [1968; 3470] N=215
Cumulative colloid amount [mL per study day]	200 [94;375] N=167	179 [92; 500] N=207	500 [200; 783] N=218
Colloid amount on day 1 [mL]	500 [500; 500] N=120	500 [500; 1000] N=135	1000 [500; 1500] N=192
Cumulative PRBC transfusion [units per study day]	0.3 [0.2; 0.6] N=76	0.3 [0.1; 0.7] N=92	0.5 [0.3; 1.0] N=101
PRBC transfusion on day 1 [units]	1.0 [0.8; 2.0] N=30	2.0 [1.9; 2.9] N=36	1.9 [1.5; 2.0] N=64
Cumulative non-PRBC transfusion [units per study day]	0.2 [0.1; 0.3] N=10	0.1 [0.1; 0.3] N=4	0.4 [0.2; 0.5] N=17
Non-PRBC transfusion on day 1 [units]	1.3 [0.5; 1.3] N=5	2.5 [2.5; 2.5] N=1	2.7 [1.5; 3.5] N=8
Cumulative urinary output [mL per study day]	2785 [2112; 3413] N=167	2653 [1972; 3345] N=207	2175 [1417; 2866] N=218
Urinary output on day 1 [mL]	2340 [1600; 33000] N=165	2315 [1500; 3200] N=200	1643 [1077; 2443] N=216

Data are given as median [25th; 75th percentiles]. N, number of patients. PBRC, packed red blood cells. Non-PRBC transfusion comprises fresh frozen plasma, lyophilised plasma, single donor platelet concentrates and multiple donor (pooled) platelet concentrates.

Supplemental digital content 5: Daily fluid balance in study patients.
The figure depicts the daily fluid balance of the study patients up to 20 days of ICU stay (blue line). Dashed lines represent 25th and 75th percentiles. A All patients, B patients receiving solely crystalloids, C patients receiving crystalloids and colloids.

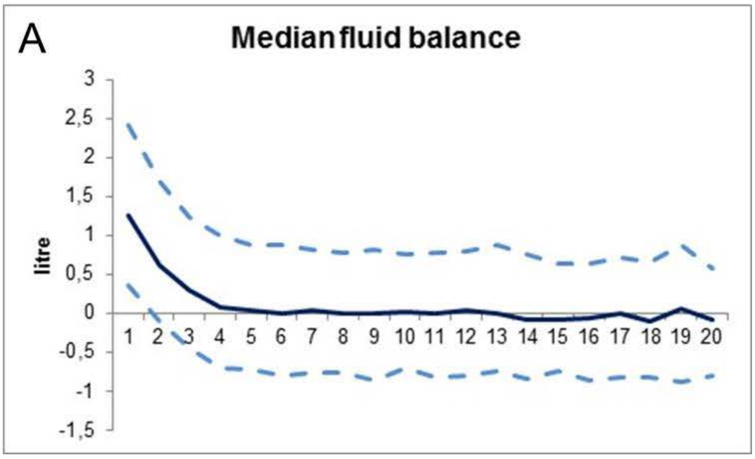

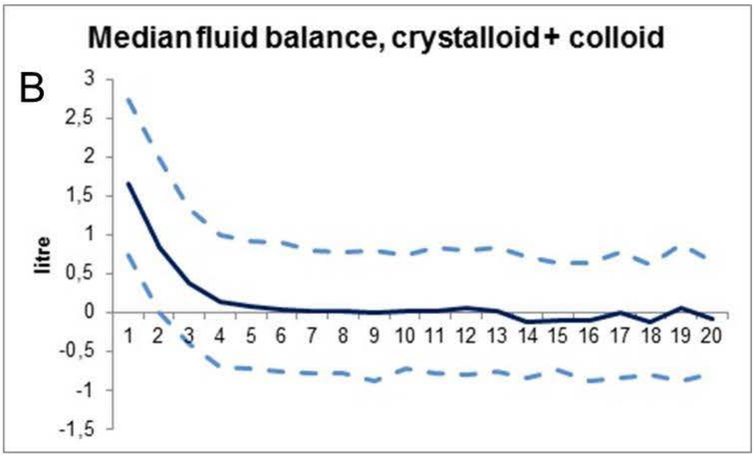

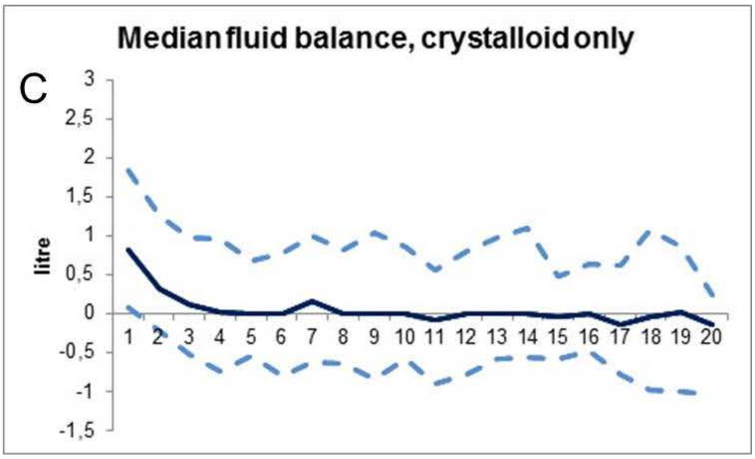

Supplemental digital content 6: Fluid balances and infusion amounts of study patients eligible for multivariable analysis and subcohorts.

	All	Crystalloids only	All colloids	p-Value[a]	HES 130/0.4	p-Value[b]
Cumulative fluid balance [mL per study day]	436 [0; 1042] N= 3902	337 [0; 945] N=1888	505 [42; 1149] N=2014	<0.001	462 [22; 1039] N=1277	<0.001
Fluid balance on day 1 [mL]	1290 [379; 2430] N=3768	762 [4;1750] N=1888	1645 [705; 2738] N=2014	<0.001	1607 [700; 2622] N=1277	<0.001
Cumulative crystalloid amount [mL per study day]	1334 [767; 2100] N=3902	1114 [526; 1844] N=1888	1518 [964; 2306] N=2014	<0.001	1395 [883; 2227] N=1277	<0.001
Crystalloid amount on day 1 [mL]	2228 [1235; 3260] N=3902	1753 [772; 2879] N=1888	2609 [1790; 3600] N=2014	<0.001	2442 [1653; 3350] N=1277	<0.001
Cumulative colloid amount [mL per study day]	250 [125; 444] N=2014	-	250 [125; 444] N=2014	-	250 [125; 400] N=1277	-
Colloid amount on day 1 [mL]	500 [500; 1000] N=1555	-	500 [500; 1000] N=1555	-	500 [500; 1000] N=1013	-
Cumulative PRBC transfusion [units per study day]	0.3 [0.2; 0.6] N=1193	0.4 [0.2; 0.7] N=248	0.3 [0.2; 0.6] N=945	0.003	0.3 [0.2; 0.6] N=580	0.012
PRBC transfusion on day 1 [units]	2.0 [1.0; 2.2] N=562	1.9 [1.0; 2.0] N=124	2.0 [1.1; 2.2] N=438	0.004	2.0 [1.1; 2.3] N=280	0.001
Cumulative non-PRBC transfusion [units per study day]	0.2 [0.1;0.4] N= 167	0.3 [0.1; 0.5] N=22	0.2 [0.1; 0.4] N=145	0.067	0.2 [0.1; 0.4] N=97	0.049
Non-PRBC transfusion on day 1 [units]	1.5 [1.0; 2.3] N=87	1.3 [1.0; 2.2] N=14	1.5 [1.0; 2.3] N=73	0.948	1.4 [1.0; 2.0] N=52	0.928
Cumulative urinary output [mL per study day]	1731 [948; 2618] N=3902	1400 [620; 2338] N=1888	2006 [1270; 2848] N=2014	<0.001	1744 [1089; 2509] N=1277	<0.001
Urinary output on day 1 [mL]	1680 [1080; 2440] N=3719	1550 [950; 2330] N=1730	1770 [1195; 2560] N=1989	<0.001	1645 [1159; 2309] N=1270	0.001

Data are given as median [25th; 75th percentiles]. N, number of patients. PBRC, packed red blood cells. Non-PRBC transfusion comprises fresh frozen plasma, lyophilised plasma, single donor platelet concentrates and multiple donor (pooled) platelet concentrates. [a] Wilcoxon-Mann-Whitney test for crystalloids vs. colloids, [b] Wilcoxon-Mann-Whitney test for crystalloids vs. HES 130/0.4

Supplemental digital content 7: Daily colloid infusion in study patients

The figure depicts the mean daily colloid dose of study patients up to 20 days of intensive care unit (ICU) stay (blue line) and the number of patients receiving colloids on successive ICU days (red line). Dashed lines represent the 25th and 75th percentiles and were constant. Notably, the median infusion amount equals the 25th percentile (500 ml) for each study day.

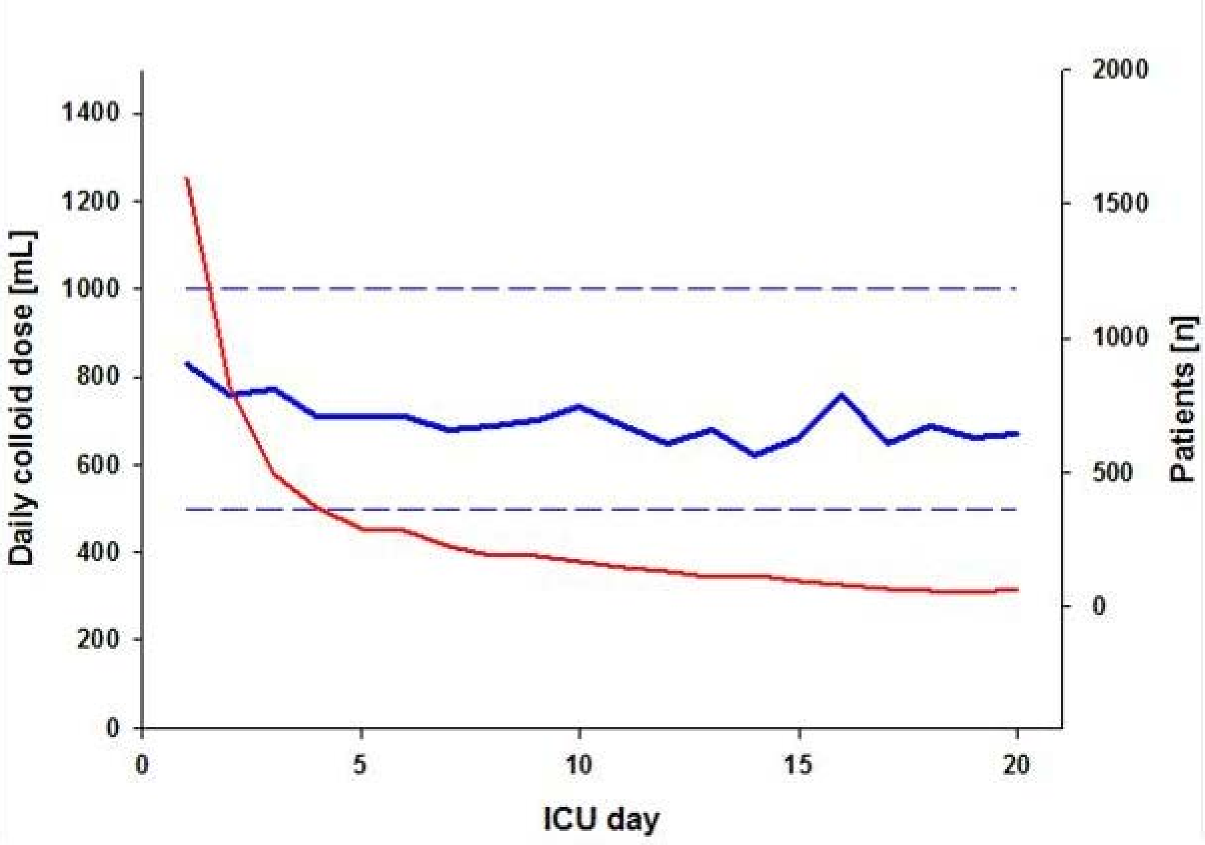

Supplemental digital content 8: Effects of colloid infusion (HES 130/0.4) in Cox regression analysis of 90-day mortality.

**Odds / Hazard ratio estimates**	**Unadjusted (only patients eligible for multivariable analysis)***			**Baseline adjustment**			**Full model**		
**Effect**	**OR**	**95% CI**		**HR**	**95% CI**		**HR**	**95% CI**	
**Colloid infusion**	**1.568**	**1.270**	**1.936**	**1.177**	**0.947**	**1.462**	**0.833**	**0.656**	**1.057**
Gender, male				1.208	0.992	1.472	1.288	1.054	1.575
Predicted risk of death 90-99%				9.155	5.056	16.578	6.342	3.405	11.814
Predicted risk of death 80-89%				5.077	2.765	9.319	3.740	2.015	6.943
Predicted risk of death 70-79%				3.827	2.086	7.022	2.775	1.495	5.151
Predicted risk of death 60-69%				3.856	2.153	6.908	3.313	1.840	5.965
Predicted risk of death 50-59%				2.237	1.181	4.240	2.008	1.058	3.813
Predicted risk of death 40-49%				2.193	1.223	3.931	1.977	1.101	3.550
Predicted risk of death 30-39%				1.865	1.031	3.375	1.744	0.963	3.158
Predicted risk of death 20-29%				1.921	1.073	3.440	1.742	0.972	3.123
Predicted risk of death 10-19%				2.185	1.230	3.881	2.085	1.173	3.706
Chronic kidney disease				2.027	1.632	2.517	3.006	2.374	3.805
Severe sepsis on admission				2.022	1.546	2.645	1.292	0.964	1.732
Vasopressor equivalent >0.6 mg/h							1.027	1.019	1.036
SOFA score AUC							1.032	1.020	1.044
Cumulative red blood cell products (per litre)							1.040	1.012	1.069
Cumulative other blood products (per litre)							0.971	0.948	0.994
Cumulative fluid balance (per litre)							1.021	0.971	1.073
Daily crystalloid infusion (per litre)							1.100	1.033	1.171

Risk of mortality was estimated from severity scores as detailed in the methods section. Gender, risk of mortality categories, vasopressor requirement, chronic kidney disease and severe sepsis on admission were used as binary variables (yes/no), whereas the hazard ratios (HR) for fluid-based variables and sequential organ failure assessment (SOFA) score describe the excess risk per litre of fluid and per SOFA area under the curve (AUC), respectively. CI, confidence interval. *To show the effect of the modelling, we showed the unadjusted OR for the population that was eligible for the multivariable analysis. Crude OR for all patients in the registry are shown in the text.

Supplemental digital content 9: Missing data analysis in Cox regression analysis of 90-day mortality for crystalloids vs. colloids

Hazard ratio estimates	Regular analysis			all patients with unknown status die 1 day after discharge			10% of all patients die after discharge (unknown status is extrapolated based on post-ICU mortality)			28% of all patients die after discharge (unknown status is extrapolated based on mortality in the cohort without 90 d follow up)			All patients with unknown status survive 90 days		
**Effect**	**HR**	**95% CI**		**HR**	**95% CI**		**HR**	**95% CI**		**HR**	**95% CI**		**HR**	**95% CI**	
Gender, male	0.798	0.679	0.936	0.803	0.722	0.892	0.775	0.667	0.901	0.803	0.700	0.922	0.798	0.679	0.936
Predicted risk of death 90-99%	4.505	2.781	7.298	1.582	1.161	2.156	3.528	2.270	5.482	2.747	1.877	4.019	4.505	2.781	7.298
Predicted risk of death 80-89%	2.630	1.637	4.226	1.118	0.835	1.496	2.284	1.492	3.497	1.563	1.072	2.280	2.630	1.637	4.226
Predicted risk of death 70-99%	2.250	1.412	3.586	1.018	0.773	1.340	1.822	1.194	2.779	1.292	0.893	1.869	2.250	1.412	3.586
Predicted risk of death 60-69%	2.184	1.403	3.401	1.082	0.846	1.385	1.843	1.239	2.740	1.451	1.039	2.028	2.184	1.403	3.401
Predicted risk of death 50-59%	1.718	1.070	2.757	1.024	0.790	1.327	1.672	1.101	2.538	1.237	0.864	1.772	1.718	1.070	2.757
Predicted risk of death 40-49%	1.732	1.128	2.660	0.973	0.779	1.216	1.586	1.086	2.316	1.217	0.887	1.669	1.732	1.128	2.660
Predicted risk of death 30-39%	1.484	0.957	2.303	0.962	0.770	1.204	1.258	0.849	1.863	1.052	0.760	1.457	1.484	0.957	2.303
Predicted risk of death 20-29%	1.611	1.045	2.482	1.018	0.818	1.266	1.490	1.018	2.181	1.192	0.870	1.635	1.611	1.045	2.482
Predicted risk of death 10-19%	1.759	1.140	2.712	1.083	0.872	1.345	1.484	1.009	2.182	1.160	0.842	1.598	1.759	1.140	2.712
Chronic kidney disease	1.960	1.645	2.336	1.356	1.196	1.537	1.768	1.496	2.089	1.755	1.505	2.047	1.960	1.645	2.336
Severe sepsis on admission	1.284	1.025	1.608	1.250	1.062	1.472	1.338	1.080	1.658	1.335	1.094	1.628	1.284	1.025	1.608
Vasopressor equivalent >0.6 mg/h	1.019	1.002	1.035	1.004	0.990	1.018	1.017	1.001	1.033	1.013	0.998	1.029	1.019	1.002	1.035
SOFA score AUC	1.030	1.024	1.036	1.011	1.007	1.015	1.026	1.020	1.031	1.021	1.015	1.026	1.030	1.024	1.036
Cumulative red blood cell products (per litre)	0.977	0.958	0.997	0.992	0.977	1.006	0.979	0.960	0.997	0.985	0.968	1.003	0.977	0.958	0.997
Cumulative other blood products (per litre)	1.002	0.968	1.037	1.014	0.984	1.044	1.007	0.974	1.042	1.005	0.973	1.039	1.002	0.968	1.037
Cumulative fluid balance (per litre)	1.014	1.008	1.020	1.005	1.000	1.010	1.011	1.004	1.017	1.008	1.002	1.014	1.014	1.008	1.020
Daily crystalloid infusion (per litre)	1.121	1.067	1.178	1.176	1.140	1.213	1.173	1.122	1.226	1.176	1.131	1.223	1.121	1.067	1.178
Daily colloid infusion (per litre)	1.003	0.980	1.027	1.000	0.981	1.020	0.997	0.974	1.020	0.994	0.971	1.017	1.003	0.980	1.027

Risk of mortality was estimated from severity scores as detailed in the methods section. Gender, risk of mortality categories, vasopressor requirement, chronic kidney disease and severe sepsis on admission were used as binary variables (yes/no), whereas the hazard ratios for fluid-based variables and sequential organ failure assessment (SOFA) score describe the excess risk per litre of fluid and per SOFA area under the curve (AUC), respectively.

Supplemental digital content 10: Effects of colloid infusion in logistic regression analysis of intensive care unit mortality.

Odds ratio estimates	Unadjusted (only patients eligible for multivariable analysis)*			Baseline adjustment			Full model		
**Effect**	**OR**	**95% CI**		**OR**	**95% CI**		**OR**	**95% CI**	
**Colloid infusion**	**3.087**	**2.416**	**3.945**	**1.547**	**1.258**	**1.903**	**0.959**	**0.919**	**1.001**
Gender, male				1.463	1.200	1.784	0.726	0.608	0.867
Predicted risk of death 90-99%				12.993	7.766	21.738	8.885	5.295	14.907
Predicted risk of death 80-89%				3.467	2.135	5.631	3.518	2.196	5.638
Predicted risk of death 70-99%				2.557	1.599	4.088	2.691	1.715	4.224
Predicted risk of death 60-69%				1.490	0.947	2.344	2.108	1.380	3.220
Predicted risk of death 50-59%				1.660	1.044	2.640	1.615	1.037	2.515
Predicted risk of death 40-49%				0.967	0.630	1.484	1.428	0.963	2.119
Predicted risk of death 30-39%				0.735	0.471	1.146	1.013	0.676	1.517
Predicted risk of death 20-29%				0.647	0.417	1.005	0.999	0.671	1.485
Predicted risk of death 10-19%				0.634	0.406	0.989	1.114	0.751	1.653
Chronic kidney disease				3.294	2.670	4.065	3.010	2.480	3.654
Severe sepsis on admission				17.298	14.008	21.361	4.917	3.960	6.104
Vasopressor equivalent >0.6 mg/h							1.130	1.090	1.171
SOFA score AUC							1.000	0.993	1.008
Cumulative red blood cell products (per litre)							0.993	0.966	1.020
Cumulative other blood products (per litre)							0.986	0.914	1.065
Cumulative fluid balance (per litre)							1.051	1.037	1.066
Daily crystalloid infusion (per litre)							0.718	0.666	0.775

Risk of mortality was estimated from severity scores as detailed in the methods section. Gender, risk of mortality categories, vasopressor requirement, chronic kidney disease and severe sepsis on admission were used as binary variables (yes/no), whereas the odds ratios (OR) for fluid-based variables and sequential organ failure assessment (SOFA) score describe the excess risk per litre of fluid and per SOFA area under the curve (AUC), respectively. CI, confidence interval. *To show the effect of the modelling, we showed the unadjusted OR for the population that was eligible for the multivariable analysis. Crude OR for all patients in the registry are shown in the text.

Supplemental digital content 11: Effects of colloid infusion (HES 130/0.4) in logistic regression analysis of intensive care unit mortality.

Odds ratio estimates	Unadjusted (only patients eligible for multivariable analysis)*			Baseline adjustment			Full model		
**Effect**	**OR**	**95% CI**		**OR**	**95% CI**		**OR**	**95% CI**	
**Colloid infusion**	**2.281**	**1.718**	**3.028**	**2.347**	**1.786**	**3.083**	**0.700**	**0.637**	**0.769**
Gender, male				1.798	1.400	2.310	0.719	0.581	0.890
Predicted risk of death 90-99%				9.124	4.992	16.675	9.714	5.364	17.593
Predicted risk of death 80-89%				3.381	1.868	6.121	4.707	2.683	8.260
Predicted risk of death 70-99%				2.201	1.219	3.974	3.019	1.743	5.229
Predicted risk of death 60-69%				1.324	0.739	2.371	2.589	1.533	4.373
Predicted risk of death 50-59%				1.294	0.700	2.390	1.531	0.875	2.679
Predicted risk of death 40-49%				0.799	0.453	1.409	1.406	0.853	2.316
Predicted risk of death 30-39%				0.719	0.403	1.283	1.088	0.656	1.804
Predicted risk of death 20-29%				0.447	0.246	0.811	1.063	0.647	1.745
Predicted risk of death 10-19%				0.530	0.294	0.955	1.188	0.728	1.938
Chronic kidney disease				3.638	2.782	4.756	3.138	2.483	3.966
Severe sepsis on admission				30.259	23.052	39.720	5.999	4.596	7.829
Vasopressor equivalent >0.6 mg/h							1.114	1.052	1.179
SOFA score AUC							0.994	0.984	1.003
Cumulative red blood cell products (per litre)							1.008	0.967	1.051
Cumulative other blood products (per litre)							1.076	0.938	1.233
Cumulative fluid balance (per litre)							1.058	1.037	1.078
Daily crystalloid infusion (per litre)							0.961	0.896	1.032

Risk of mortality was estimated from severity scores as detailed in the methods section. Gender, risk of mortality categories, vasopressor requirement, chronic kidney disease and severe sepsis on admission were used as binary variables (yes/no), whereas the odds ratios (OR) for fluid-based variables and sequential organ failure assessment (SOFA) score describe the excess risk per liter of fluid and per SOFA area under the curve (AUC), respectively. CI, confidence interval. *To show the effect of the modelling, we showed the unadjusted OR for the population that was eligible for the multivariable analysis. Crude OR for all patients in the registry are shown in the text.

Supplemental digital content 12: Effects of colloid infusion (HES 130/0.4) in logistic regression analysis of RIFLE "failure".

Odds ratio estimates	Unadjusted (only patients eligible for multivariable analysis)*			Baseline adjustment			Full model		
**Effect**	**OR**	**95% CI**		**OR**	**95% CI**		**OR**	**95% CI**	
**Colloid infusion**	**1.931**	**1.541**	**2.419**	**2.260**	**1.730**	**2.953**	**0.800**	**0.704**	**0.910**
Gender, male				1.248	0.977	1.594	0.975	0.707	1.343
Predicted risk of death 90-99%				23.968	11.937	48.123	3.797	0.501	28.792
Predicted risk of death 80-89%				6.825	3.426	13.596	3.839	0.505	29.163
Predicted risk of death 70-99%				5.622	2.882	10.969	2.882	0.377	22.010
Predicted risk of death 60-69%				3.575	1.869	6.839	5.173	0.693	38.624
Predicted risk of death 50-59%				3.211	1.634	6.312	3.965	0.515	30.554
Predicted risk of death 40-49%				1.729	0.914	3.271	2.460	0.321	18.886
Predicted risk of death 30-39%				1.808	0.951	3.438	2.348	0.300	18.357
Predicted risk of death 20-29%				0.947	0.490	1.830	2.067	0.257	16.652
Predicted risk of death 10-19%				1.261	0.660	2.407	1.965	0.204	18.972
Chronic kidney disease				13.279	10.221	17.251	2.577	1.861	3.569
Severe sepsis on admission				10.813	8.412	13.900	0.791	0.475	1.318
Vasopressor equivalent >0.6 mg/h							0.860	0.763	0.969
SOFA score AUC							1.070	1.060	1.080
Cumulative red blood cell products (per litre)							0.805	0.620	1.046
Cumulative other blood products (per litre)							1.015	0.956	1.079
Cumulative fluid balance (per litre)							0.995	0.963	1.027
Daily crystalloid infusion (per litre)							1.225	1.115	1.347

Risk of mortality was estimated from severity scores as detailed in the methods section. Gender, risk of mortality categories, vasopressor requirement, chronic kidney disease and severe sepsis on admission were used as binary variables (yes/no), whereas the odds ratios (OR) for fluid-based variables and sequential organ failure assessment (SOFA) score describe the excess risk per liter of fluid and per SOFA area under the curve (AUC), respectively. CI, confidence interval. *To show the effect of the modelling, we showed the unadjusted OR for the population that was eligible for the multivariable analysis. Crude OR for all patients in the registry are shown in the text.

Supplemental digital content 13: Urine output and creatinine concentrations

Supplemental digital content 13A:Median creatinine (blue line) and quartiles (dashed lines).

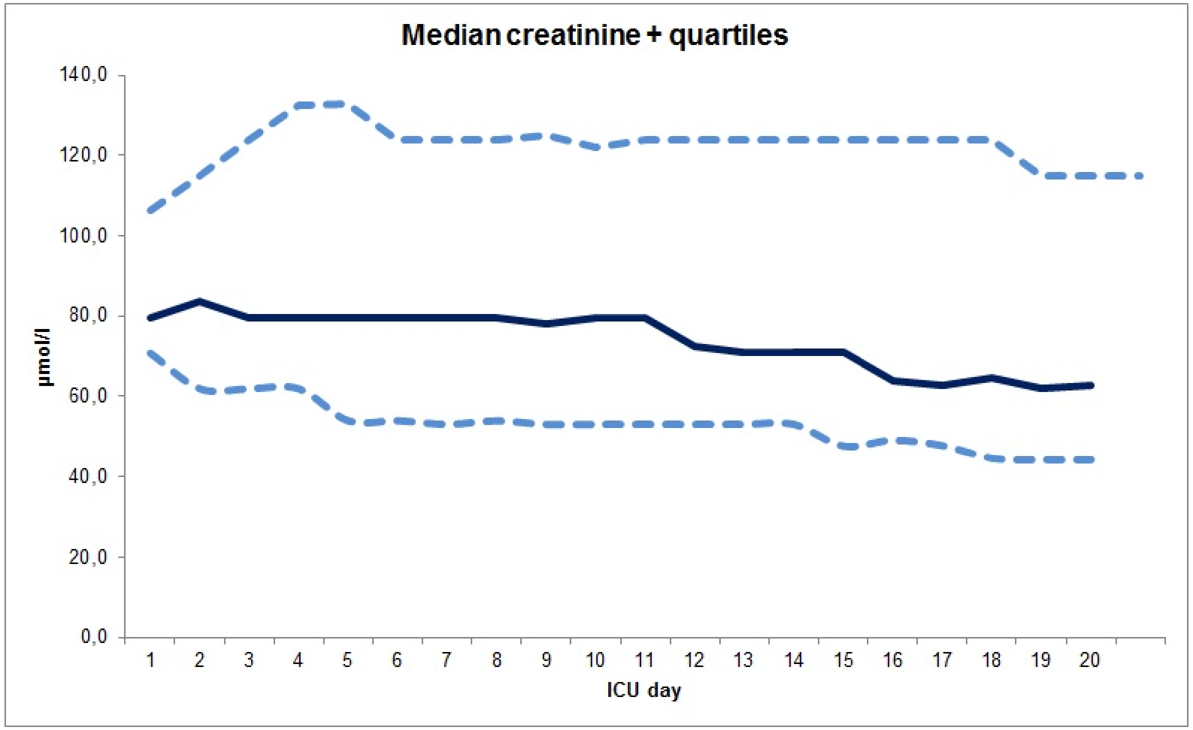

Supplemental digital content 13B: Urine output. Median (blue line) and 25th and 75th percentile (dashed lines).

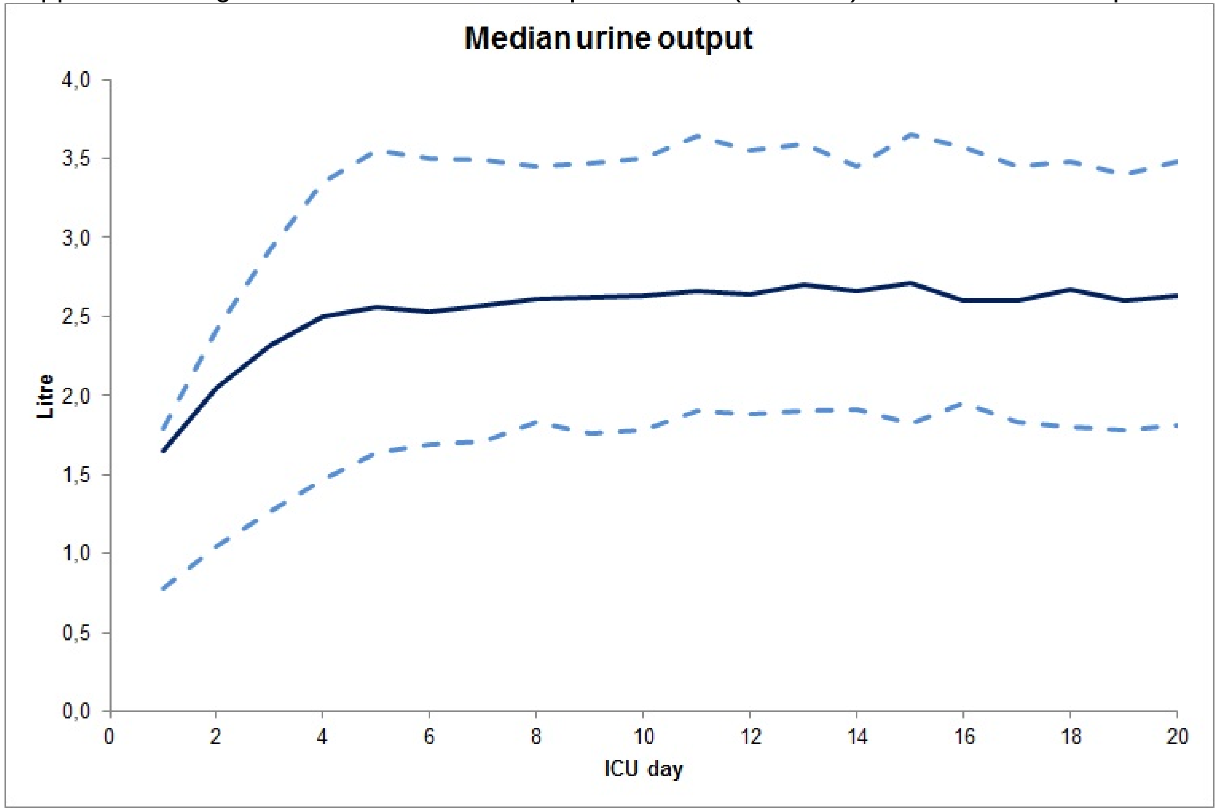

Supplemental digital content 14: Acute kidney injury in study patients with or without pre-existing chronic kidney disease (CKD) on admission.
Data are given as n (%).

	All	RIFLE „failure“		Renal replacement therapy	
		**Yes**	**No**	**Yes**	**No**
CKD on admission					
Yes	824 (100.0)	324 (39.3)	500 (60.7)	194 (23.5)	630 (76.5)
No	3721 (100.0)	236 (6.3)	3485 (93.7)	167 (4.5)	3554 (95.5)

Supplemental digital content 15: Effects of colloid infusion in logistic regression analysis of renal replacement therapy.

Supplemental digital content 15A: Effects of colloid infusion in logistic regression analysis of renal replacement therapy.

**Odds ratio estimates**	**Unadjusted (only patients eligible for multivariable analysis)***			**Baseline adjustment**			**Full model**		
**Effect**	**OR**	**95% CI**		**OR**	**95% CI**		**OR**	**95% CI**	
**Colloid infusion**	**3.882**	**2.988**	**5.043**	**1.953**	**1.569**	**2.431**	**0.877**	**0.790**	**0.973**
Gender, male				1.082	0.878	1.334	0.985	0.786	1.234
Predicted risk of death 90-99%				17.761	10.359	30.451	12.614	6.804	23.387
Predicted risk of death 80-89%				2.896	1.728	4.853	2.928	1.636	5.239
Predicted risk of death 70-99%				2.414	1.473	3.957	2.260	1.298	3.935
Predicted risk of death 60-69%				1.414	0.880	2.271	1.388	0.818	2.355
Predicted risk of death 50-59%				1.373	0.839	2.248	1.444	0.842	2.476
Predicted risk of death 40-49%				0.951	0.608	1.490	1.105	0.675	1.807
Predicted risk of death 30-39%				0.597	0.370	0.961	0.712	0.426	1.188
Predicted risk of death 20-29%				0.573	0.360	0.913	0.626	0.377	1.040
Predicted risk of death 10-19%				0.698	0.441	1.107	0.816	0.494	1.346
Chronic kidney disease				7.685	6.198	9.528	6.135	4.889	7.698
Severe sepsis on admission				14.653	11.817	18.169	14.881	11.542	19.186
Vasopressor equivalent >0.6 mg/h							0.779	0.731	0.830
SOFA score AUC							1.048	1.040	1.056
Cumulative red blood cell products (per litre)							0.997	0.951	1.046
Cumulative other blood products (per litre)							1.145	0.933	1.405
Cumulative fluid balance (per litre)							1.037	1.017	1.057
Daily crystalloid infusion (per litre)							0.682	0.626	0.742

Risk of mortality was estimated from severity scores as detailed in the methods section. Gender, risk of mortality categories, vasopressor requirement, chronic kidney disease and severe sepsis on admission were used as binary variables (yes/no), whereas the odds ratios (OR) for fluid-based variables and sequential organ failure assessment (SOFA) score describe the excess risk per liter of fluid and per SOFA area under the curve (AUC), respectively. CI, confidence interval. *To show the effect of the modelling, we showed the unadjusted OR for the population that was eligible for the multivariable analysis. Crude OR for all patients in the registry are shown in the text.

Supplemental digital content 15B: Effects of HES130/0.4 infusion in logistic regression analysis of renal replacement therapy.

**Odds ratio estimates**	**Unadjusted (only patients eligible for multivariable analysis)***			**Baseline adjustment**			**Full model**		
**Effect**	**OR**	**95% CI**		**OR**	**95% CI**		**OR**	**95% CI**	
**Colloid infusion**	**3.187**	**2.379**	**4.269**	**3.465**	**2.591**	**4.632**	**0.509**	**0.441**	**0.586**
Gender, male				1.494	1.151	1.939	0.708	0.528	0.949
Predicted risk of death 90-99%				12.384	6.554	23.401	16.016	7.282	35.226
Predicted risk of death 80-89%				3.039	1.617	5.711	5.238	2.431	11.284
Predicted risk of death 70-99%				2.445	1.325	4.513	3.286	1.559	6.928
Predicted risk of death 60-69%				1.193	0.648	2.197	1.700	0.825	3.501
Predicted risk of death 50-59%				1.278	0.675	2.419	1.700	0.804	3.596
Predicted risk of death 40-49%				0.717	0.395	1.304	1.193	0.593	2.400
Predicted risk of death 30-39%				0.557	0.297	1.045	0.882	0.429	1.812
Predicted risk of death 20-29%				0.415	0.221	0.780	0.610	0.294	1.264
Predicted risk of death 10-19%				0.581	0.315	1.073	0.904	0.445	1.839
Chronic kidney disease				9.588	7.250	12.680	6.987	5.173	9.438
Severe sepsis on admission				21.657	16.497	28.431	22.874	16.149	32.398
Vasopressor equivalent >0.6 mg/h							0.633	0.568	0.705
SOFA score AUC							1.064	1.052	1.076
Cumulative red blood cell products (per litre)							0.987	0.905	1.077
Cumulative other blood products (per litre)							0.927	0.684	1.257
Cumulative fluid balance (per litre)							1.030	0.995	1.066
Daily crystalloid infusion (per litre)							1.084	0.934	1.257

Risk of mortality was estimated from severity scores as detailed in the methods section. Gender, risk of mortality categories, vasopressor requirement, chronic kidney disease and severe sepsis on admission were used as binary variables (yes/no), whereas the odds ratios (OR) for fluid-based variables and sequential organ failure assessment (SOFA) score describe the excess risk per liter of fluid and per SOFA area under the curve (AUC), respectively. CI, confidence interval. *To show the effect of the modelling, we showed the unadjusted OR for the population that was eligible for the multivariable analysis. Crude OR for all patients in the registry are shown in the text.
